# Prior term delivery increases risk of subsequent recurrent preterm birth: An unexpected finding

**DOI:** 10.1111/ajo.13504

**Published:** 2022-02-27

**Authors:** Natalie Suff, Vicky X. Xu, Giorgia Dalla Valle, Jenny Carter, Shaun Brennecke, Andrew Shennan

**Affiliations:** ^1^ 4616 Department of Women and Children's Health School of Life Course Sciences Faculty of Life Sciences and Medicine King's College London London UK; ^2^ 2541 Medicine Department, Nursing and Health Sciences Monash University Melbourne Victoria Australia; ^3^ University of Melbourne Department of Obstetrics and Gynaecology Royal Women’s Hospital Melbourne Victoria Australia; ^4^ Pregnancy Research Centre Department of Maternal‐Fetal Medicine Royal Women's Hospital Melbourne Victoria Australia

**Keywords:** preterm birth, caesarean section, obstetrics, late miscarriage

## Abstract

**Background:**

Women with a prior pregnancy at term are generally considered to be at reduced risk for subsequent spontaneous preterm birth (sPTB), whereas a previous sPTB is a major predictor for a future sPTB.

**Aims:**

The objective of this study was to investigate the risk of recurrent sPTB in women with a prior term birth and a subsequent sPTB.

**Materials and Methods:**

This is a retrospective cohort study conducted at St Thomas’ Hospital in London, UK. There were 430 women included: 230 with a term birth (caesarean section or vaginal delivery) preceding a sPTB (term + sPTB group) and 200 with a prior sPTB only (sPTB only group). The primary outcome was sPTB, <37 weeks gestation.

**Results:**

Of the term + sPTB group, 38.7% (89/230) had a recurrent sPTB compared to 20% (40/200) in the sPTB only group (*P* < 0.0001), with a relative risk (RR) of 1.9. Of women who had a term caesarean section and a subsequent PTB, 50% (30/60) had a further sPTB (RR 2.5 compared to the sPTB only group), while 34.7% (59/170) of women who had a term vaginal birth and subsequent sPTB, had a further sPTB (RR 1.7 compared to the sPTB only group).

**Conclusion:**

In women who have had a previous sPTB, the risk of a recurrence is much higher than in women with a prior term birth. The aetiology of PTB may be different in this subgroup of women and needs to be further elucidated to determine how best to identify and treat them.

## INTRODUCTION

Preterm birth (PTB) is a global health concern, and the leading cause of perinatal mortality and morbidity.^1^, ^2^ Despite many publications on the subject, the aetiology of spontaneous PTB (sPTB) remains largely undefined.^3^, ^4^ Although the major predictor for sPTB is a prior sPTB, in some cases PTB occurs after a previous birth at term.^5^ Women with a pregnancy at term are generally considered to be at reduced risk for subsequent PTB; however, specific risk factors including short interpregnancy interval and new tobacco use have been implicated.^6^

A significant association between emergency caesarean sections (EmCS) at term and sPTB in subsequent pregnancies has been reported in recent years, with as much as a six‐fold increased risk of sPTB following a full dilatation EmCS.^7^, ^8^, ^9^, ^10^ More worrying is the high risk of a recurrent sPTB in this group of women.^11^ Recent evidence suggests that the risk of a recurrent sPTB following an initial term full dilatation EmCS could be up to three times the risk of that following an initial term vaginal birth (VD).^11^, ^12^ Therefore, the objective of this study was to evaluate the risk of sPTB recurrence following an initial term birth.

## MATERIALS AND METHODS

This was an observational, retrospective cohort study conducted at St Thomas’ Hospital in London, United Kingdom. All participants were identified through the Preterm Clinical Network (PCN) Database (Research Ethics Service (REC) Ref. 16/ES/0093).^13^

Our study group all had a prior term birth followed by a subsequent PTB. Eligible women in this group (term + sPTB group) required three consecutive pregnancies: a term pregnancy (VD or CS) (Pregnancy A), followed by a sPTB (Pregnancy B), and a subsequent index pregnancy. Our control group (sPTB only group) consisted of women who had two consecutive pregnancies: a sPTB by VD (Pregnancy A), and a subsequent index pregnancy. Pregnancies that reached at least 14 weeks gestation were included. Women were excluded if they had: any pregnancy >14 weeks gestation prior to pregnancy A in either group, a termination of pregnancy (for fetal anomalies or social indications), an iatrogenic preterm birth, treatment with a transabdominal cerclage or a multiple pregnancy in any of their pregnancies. Women who had a transvaginal cerclage, the preventative treatment option provided in this unit, in any pregnancy were included. Demographic characteristics are those at the time of the index pregnancy.

The primary outcome was sPTB <37 weeks gestation in the index pregnancy. sPTB included: women with spontaneous onset of labour; women with preterm prelabour rupture of membranes; women who had a spontaneous midtrimester loss between 14–24 weeks; or women who presented with signs of cervical insufficiency (eg cervical dilation, bulging membranes) followed by intrauterine death requiring induction of labour.

Secondary outcomes included sPTB further categorised by gestational ages: <32 weeks and <24 weeks gestation. Women in the prior term birth group were also subcategorised into EmCS and VD to evaluate their respective outcomes in comparison to control women.

Data were expressed as median + interquartile range (IQR) for continuous variables and percentage for categorical variables. Univariate logistic regression was used to analyse the primary outcome. The rate of sPTBs was translated to a relative risk (RR) with a confidence interval (CI) of 95%. To ascertain the primary and secondary outcomes, χ^2^ tests were used. The control group (no prior term birth) was the reference group against which RRs were calculated. Testing for differences of non‐parametric variables (gestational age at birth) was accomplished using Kruskal‐Wallis tests to compare the three groups and Wilcoxon matched‐pair signed rank test for the comparison of paired sPTB gestational ages. Comparison between the groups for categorical variables was performed using the χ^2^ test. The Kaplan–Meier plot with log‐rank (Mantel‐Cox) test were used for comparison of gestational age at birth. The association of sPTB (<37 weeks gestation) with prior term + sPTB and identifiable risk factors for sPTB (other than prior sPTB) was assessed via Cox regression analysis. A *P*‐value of <0.05 was considered to signify statistical significance. GraphPad Prism 9 (GraphPad Software, San Diego, CA, USA) was used for statistical analysis.

This project was undertaken under the ethics approval for the PCN Database, under REC Ref. 16/ES/0093 by the East of Scotland REC.^13^

## RESULTS

All demographic characteristics were similar between the term+sPTB group and the sPTB only group, except for ethnicity and current smoker status (Table 1).

**Table 1:** Maternal demographics comparing the term + sPTB group with the control group, sPTB only group

Demographics		Term + sPTB (*N* = 230)	sPTB only (*N* = 200)
Age, mean (SD)		31.5 (5.5)	31.9 (5.3)
BMI, mean (SD)		27.9 (6.2)	26.8 (6.4)
Ethnicity	European	33.5% (77)	49.5% (99)
	Other	3.0% (7)	4.5% (9)
	Asian	6.5% (15)	7.5% (15)
	African/Afro‐Caribbean	57.0% (131)	38.5% (77)
Cervical surgery prior to index		8.7% (20)	10% (20)
Smoking current		13.2% (30/228)	4% (8)
History	Ex	10.1% (23/228)	15.5% (31)
	Never	76.7% (175/228)	80.5% (161)

BMI, body mass index; sPTB, spontaneous preterm birth; SD, standard deviation.

**Figure 1:**
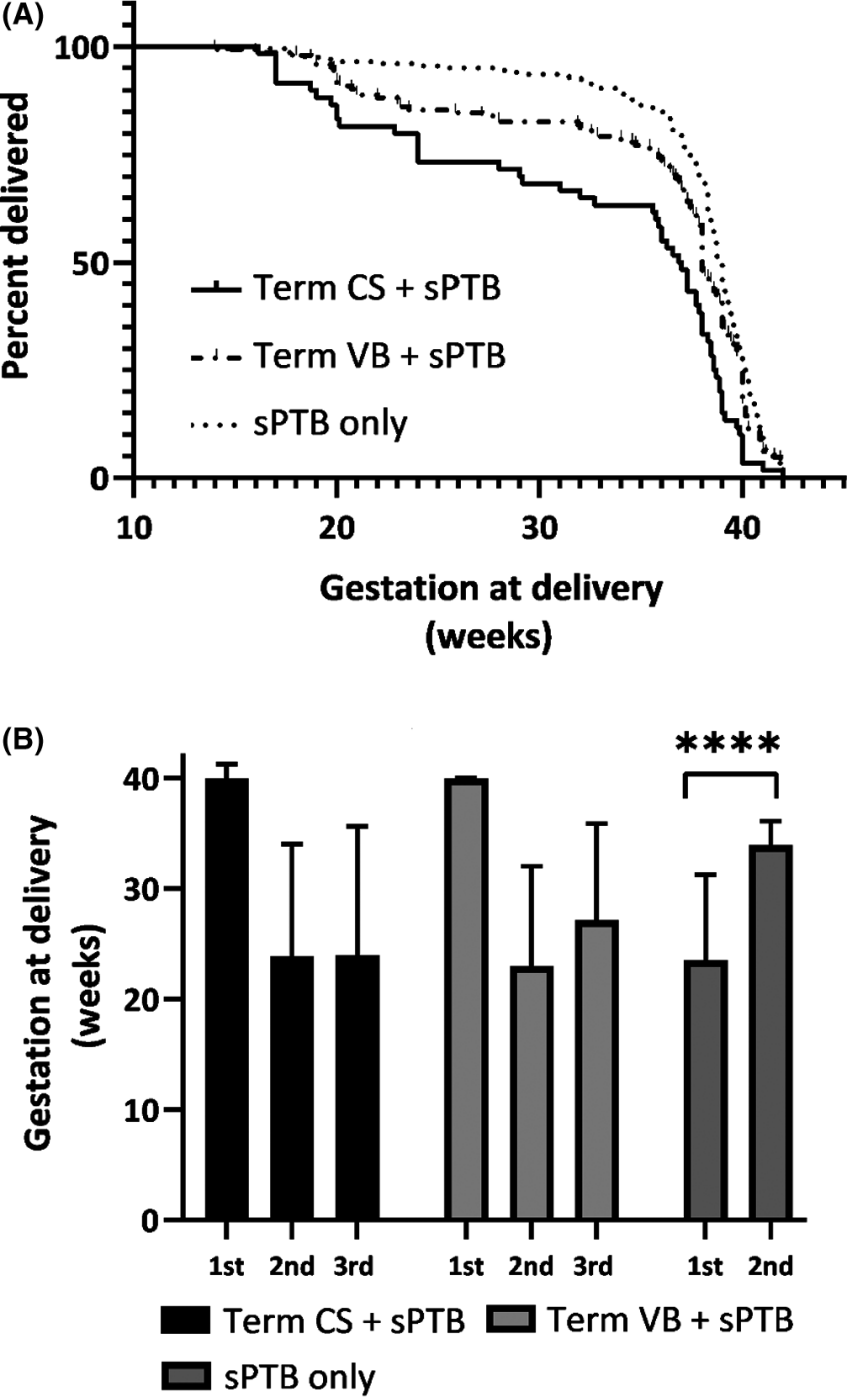
Gestational age at birth among the three groups (term CS + sPTB, term VB + sPTB and sPTB only groups) (A). The gestational age at birth of women who delivered preterm (<37 weeks gestation) in the index pregnancy is significantly higher compared to the first sPTB in the sPTB only group (B). A: Kaplan–Meier curve of gestational age of birth in the index pregnancy comparing term CS + sPTB, term VB + sPTB and sPTB only control women. ****P < 0.0001 on log‐rank (Mantel‐Cox) test. B: Bar chart of median gestational ages at birth of women who delivered preterm (<37 weeks gestation) in the 1st sPTB and index pregnancy comparing term CS + sPTB, term VB + sPTB and sPTB only, with a table showing median (+IQR) gestational ages at birth of sPTB (1st and 2nd) in each group. ****P < 0.0001 on Mann‐Whitney test, comparing the gestational age at birth of the 1st and 2nd sPTB within each group. CS, caesarean section; IQR, interquartile range; sPTB, spontaneous preterm birth; RF, risk factor; VB, vaginal birth.

Of term + sPTB women, 38.7% (89/230) had a further sPTB < 37 weeks gestation in the index pregnancy compared to 20% (40/200) in the sPTB only women (*P* < 0.0001; Table 2). By further separating prior term deliveries into CS and VD, 50% (30/60) of women with a term CS + sPTB had a further sPTB <37 weeks gestation, compared with 34.7% (59/170) of women who had a term VD + sPTB (*P* < 0.0001; Table 2). Of the term CS + sPTB group only 8% (5/60) were elective CS, the remaining 92% (55/60) were emergency CS with 33% (18/55) being full dilatation CS. The median gestational age of birth in women with a term CS + sPTB was 36.9 weeks gestation and 38.0 weeks gestation in the term VD + sPTB group, compared with 38.9 weeks gestation in the sPTB only group of women (*P* < 0.0001; Table 2). Kaplan–Meier ‘Survival’ estimates depict the proportion of women undelivered at each gestational week according to term CS + sPTB, term VD + sPTB and sPTB only controls (*P* < 0.0001, Fig. 1A). Compared to the sPTB only controls, term+sPTB was associated with a significantly higher rate of sPTB using Cox regression analysis, after adjusting for identifiable risk factors for sPTB (other than prior sPTB), with an adjusted hazard ratio of 2.3 (95% CI = 1.6–3.4).

**Table 2:** Gestational age at birth, rates of recurrent sPTB and relative risks (RR) of recurrent PTB in women in the term + sPTB group (subcategorised into CS and VD) compared with the sPTB only group

PTB rates by mode of birth in the term pregnancy	Term + sPTB		sPTB only	*P*‐value			
	Term CS	Term VD	No term birth				
Gestational age at birth in index pregnancy median (IQR)	36.9 (24–38.7)	38.0 (35.1–40.0)	38.9 (37.3–40.1)	<0.0001			
PTB <37 weeks gestation	38.7% (89/230)		20% (40/200)	<0.0001			
	50% (30/60)	34.7% (59/170)					
PTB <37 weeks RR [95% CI]^†^	2.5 [1.7–3.6]	1.7 [1.2–2.5]	1.0	<0.0001			0.002
PTB <32 weeks gestation	26.1% (60/230)		7% (14/200)	<0.0001			
	33.3% (20/60)	20% (34/170)					
PTB <32 weeks RR [95% CI]^†^	4.8 [2.6–8.7]	2.9 [1.6–5.1]	1.0	<0.0001		0.0003	
PTB <24 weeks gestation	17.4% (40/230)		4.5% (9/200)	<0.0001			
	20% (12/60)	14.1% (24/170)					
PTB <24 weeks RR [95% CI]^†^	4.4 [2.0–9.8]	3.1 [1.5–6.5]	0.1	0.0004	0.0016		

^†^Using the control group, sPTB only, as reference for RR.

CS, caesarean section; CI, confidence interval; IQR, interquartile range; (s)PTB, (spontaneous) preterm birth; VD, vaginal delivery.

The RR of a recurrent sPTB <37 weeks gestation for women in the term CS + sPTB group was 2.5 (95% CI 1.7–3.6) compared with the sPTB only group, and was 1.7 (95% CI 1.2–2.5) in the term VD + sPTB group compared with the sPTB only group (Table 2). For sPTB and midtrimester loss <24 weeks gestation, the RR was even higher, at 4.4 for women with a term CS + sPTB (95% CI 2.0–9.8; Table 2) and 3.1 for women with term VD + sPTB (95% CI 1.5–6.5; Table 2).

Known risk factors for sPTB were determined in each group to explore the differences in sPTB recurrence. As expected, the term CS + sPTB group had a significantly higher number of women with an identifiable risk factor (43.3%, 13/30); full dilatation CS accounted for the majority of cases. However, despite the significantly higher rates of sPTB recurrence in the term VD + sPTB group compared with the sPTB only group (34.7% vs 20%), there was no difference in identifiable risk factors (8.5% vs 10%) (Table 3). In addition, there was no significant difference in cervical cerclage use between the groups, although there may be a trend toward more cervical cerclage use in the term + sPTB group (16% in the term + sPTB group vs 10% in the sPTB only group, *P *= 0.07) (Table 3). However, there were more significant numbers of emergency rescue cerclages in the sPTB only group, compared with the term + sPTB group (24% in the term + sPTB group vs 55% in the sPTB only group, *P* = 0.04) (Table 3).

**Table 3:** Identifiable RFs for sPTB and interventions in women in the term + sPTB group (subcategorised into CS and VD) compared with the sPTB only group

	Term CS + sPTB	Term VD + sPTB	sPTB only	*P*‐value
Identifiable RF other than previous PTB (cervical surgery, full dilatation CS, uterine anomaly)	43.3% (13/30)	8.5% (5/59)	10% (4/40)	<0.0001
Prior operative VD in 1st pregnancy	0% (0/30)	6.8% (7/59)	5% (2/40)	*0.09*
Cervical cerclage in index pregnancy				
All types	16%(37/230)		10% (20/200)	*0.07*
	18.3% (11/60)	15.3% (26/170)		
Elective	48.6% (18/37)		30% 6/20	0.05
	54.5% (6/11)	46% (12/26)		
Ultrasound‐ indicated (incl. rescue)	51.4% 19/37		70% 14/20	0.26
	45.5% (5/11)	54% (14/26)		
Rescue	24% (9/37)		55% (11/20)	0.04
	36% (4/11)	19% (5/26)		
PTB <37 weeks despite cervical cerclage	45.9% (17/37)		20% (4/20)	0.08
	54.5% (6/11)	42% (11/26)		
PTB <37 weeks following rescue cerclage	54.5% (6/11)		100% 4/4	0.23
	67% (4/6)	40% (2/5)		

CS, caesarean section; (s)PTB, (spontaneous) preterm birth; RF, risk factors; VD, vaginal delivery.

Interestingly, we found that by comparing gestational age at birth between the first sPTB and the second sPTB in women who experienced a recurrent sPTB, there was a significantly longer gestational age at birth of recurrent/second sPTB in those women who did not have a prior term birth (24.0 weeks vs 34.6 weeks gestation; *P* < 0.001 Fig. 1B). In those women with a prior term birth, the recurrent sPTB occurred at a similar gestation (term CS + sPTB; 23.9 vs 24.0 weeks gestation and term VD + sPTB; 23 vs 27.1 weeks gestation, *P* = 0.7 and *P* = 0.09 respectively, Fig. 1b).

## DISCUSSION

In women who had a prior term birth by CS or VD and had a subsequent sPTB, 38.7% had a further PTB, compared to 20% in women who had no prior term birth. The focus on recurrent sPTB in this study was a novel approach to this topic. These findings can partly be explained by a CS birth, specifically those performed at full dilatation, which has been shown to increase the risk of subsequent and recurrent PTB. However, we have shown that in women with prior term VD, the risk of a recurrent sPTB is still higher than in those without a prior term birth (34.7% vs 20%). In the context of current knowledge on the main risk factors for sPTB including cervical surgery, smoking status and uterine anomalies, this difference in recurrence cannot be explained. However, our findings suggest that the aetiological cause of PTB is different and potent as it results in an earlier PTB, that is more likely to recur.

Prior term VDs are generally considered to be protective against future PTBs, with sPTB risk reported to be approximately 5–7% in subsequent pregnancies. However, in the group of women who do have a sPTB after a term birth, little is known about the recurrence rates. Data from Korea have shown that a recurrence rate of a PTB in a third pregnancy was more closely associated with women having a history of preterm birth in the second pregnancy rather than in the first pregnancy.^14^ Data from a study in 1985 further supports this; they show that the risk of another sPTB following an initial sPTB is 15.4%, yet this increases to 23.4% if there was a prior term birth.^15^ Furthermore, it has been shown that the earlier the gestation at birth in the last PTB the higher the likelihood of a recurrence.^16^ This may explain why in women in the prior term birth group who have had a recurrent sPTB, their first sPTB tended to occur extremely preterm. The circumstances surrounding birth may have an impact on the risk of subsequent PTB. CS in labour has recently been associated with PTB risk and it is thought that inadvertently low‐positioned or a caudally extended caesarean hysterotomy could damage the cervix and increase the risk of cervical insufficiency.^17^, ^18^ Likewise, distension of the cervix by the fetal head during a prolonged second stage could theoretically cause cervical damage regardless of the eventual mode of birth.^19^ The length of the second stage would be an important data point to include in future prospective studies.

A high proportion of the recurrent PTBs in the term+sPTB group are midtrimester losses, occurring at gestations < 24 weeks. The reasons for this are unknown but the postulated mechanism of cervical damage occurring during a previous birth may lead to more severe cervical insufficiency in subsequent pregnancies which result in extreme prematurity and/or loss.

Recent data have shown that women who have episodes of threatened preterm labour (TPTL) followed by a term birth, are at higher risk of subsequent PTB.^20^ The mechanism for these associations is unclear, and unfortunately, we do not have any information on TPTL episodes in our cohort. However, these results suggest that the causal factors that contribute to TPTL are potentially active in a subsequent pregnancy, so could potentially identify women who are predisposed to sPTB.

Why there is a significant difference in gestational age between the first and second PTB in women without a prior term birth is unclear and cannot be explained by higher rates of preventative therapies in this group (cervical cerclage rates were similar between the groups, with a trend toward more elective history‐indicated cerclages in the term + sPTB group). Furthermore, rates of rescue cerclage, which are much more likely to fail and be associated with an earlier birth, were higher in the sPTB only group. However, it is possible that the mechanisms behind prematurity in the women with a prior term birth may mean that their risk cannot be determined by conventional cervical length screening.

This study has also shown that approximately half of women in the prior term birth group still deliver preterm despite a cervical cerclage, compared with only 20% in our sPTB only group although these numbers are small. This is consistent with previous published data showing that transvaginal cerclage is less effective at preventing PTB in women who have a sPTB after previous term EmCS compared with other high‐risk women.^12^

Although our data suggest a causative link between a prior term birth and subsequent recurrent sPTB, prospective confirmation would be important and is the subject of ongoing investigation.^21^ Data in this current study spanned from 2000 to 2020, and over these 20 years, clinical practice has significantly evolved. CSs performed two decades ago are likely to have used different techniques from the present (eg, suture use and uterine closure techniques) which may influence outcomes. As a retrospective audit of women who had attended this institution’s high‐risk PTB surveillance clinic, there are inherent biases. However, demographics and potential confounders between prior term and no prior term birth women were shown not to have significantly affected outcomes. It is also possible that some women may have acquired new risk factors for sPTB following their term birth that we were unable to account for in this study. These could include new tobacco use and other changes in lifestyle which may affect body mass index and stress/anxiety levels. With these limitations in mind, this topic of prior term birth and recurrent sPTB would benefit from a prospective study. As this study was focused on the risk of recurrence of PTB, additional risk factors were unlikely to introduce bias.

Our study suggests that a prior term birth, by CS or VD, is associated with a significantly increased risk of recurrent sPTB. It is imperative that further work determines which aetiological factors contribute to PTB in this cohort. In addition, it is important to explore how best to offer clinical surveillance and preventative therapies in this cohort. Prior term deliveries are traditionally viewed as reassuring but in the context of a subsequent PTB they should be considered as a significant risk factor. An ongoing multi‐centre prospective study in the UK will strengthen our knowledge on the relationship between term EmCS and subsequent sPTB.^21^

## Funding

Tommy’s Baby Charity.
